# Human resource constraints and the prospect of task-sharing among community health workers for the detection of early signs of pre-eclampsia in Ogun State, Nigeria

**DOI:** 10.1186/s12978-016-0216-y

**Published:** 2016-09-30

**Authors:** David O. Akeju, Marianne Vidler, J. O. Sotunsa, M. O. Osiberu, E. O. Orenuga, Olufemi T. Oladapo, A. A. Adepoju, Rahat Qureshi, Diane Sawchuck, Olalekan O. Adetoro, Peter von Dadelszen, Olukayode A. Dada, the CLIP Nigeria Feasibility Working Group

**Affiliations:** 1 Department of Sociology, University of Lagos, Lagos, Nigeria; 2 Department of Obstetrics and Gynaecology, and the Child and Family Research Unit, University of British Columbia, Vancouver, V5Z 4H4 Canada; 3 Department of Obstetrics and Gynaecology, Babcock University Teaching Hospital, Ilishan-Remo, Nigeria; 4 Centre for Research in Reproductive Health, Sagamu, Nigeria; 5 Division of Women and Child Health, Aga Khan University, Karachi, Pakistan; 6 Department of Research, Vancouver Island Health Authority, Victoria, V8R 1 J8 Canada; 7 Department of Obstetrics and Gynaecology, OlabisiOnabanjo University, Sagamu, Nigeria; 8 Department of Obstetrics and Gynaecology, St George’s University London, London, SW17 0RE UK

**Keywords:** Task-sharing, Pre-eclampsia, Community health extension workers, Nigeria

## Abstract

**Background:**

The dearth of health personnel in low income countries has attracted global attention. Ways as to how health care services can be delivered in a more efficient and effective way using available health personnel are being explored. Task-sharing expands the responsibilities of low-cadre health workers and allows them to share these responsibilities with highly qualified health care providers in an effort to best utilize available human resources. This is appropriate in a country like Nigeria where there is a shortage of qualified health professionals and a huge burden of maternal mortality resulting from obstetric complications like pre-eclampsia. This study examines the prospect for task-sharing among Community Health Extension Workers (CHEW) for the detection of early signs of pre-eclampsia, in Ogun State, Nigeria.

**Methods:**

This study is part of a larger community-based trial evaluating the acceptability of community treatment for severe pre-eclampsia in Ogun State, Nigeria. Data was collected between 2011 and 2012 using focus group discussions; seven with CHEWs (*n* = 71), three with male decision-makers (*n* = 35), six with community leaders (*n* = 68), and one with member of the Society of Obstetricians and Gynaecologists of Nigeria (*n* = 9). In addition, interviews were conducted with the heads of the local government administration (*n* = 4), directors of planning (*n* = 4), medical officers (*n* = 4), and Chief Nursing Officers (*n* = 4). Qualitative data were analysed using NVivo version 10.0 3 computer software.

**Results:**

The non-availability of health personnel is a major challenge, and has resulted in a high proportion of facility-based care performed by CHEWs. As a result, CHEWs often take on roles that are designated for senior health workers. This role expansion has exposed CHEWs to the basics of obstetric care, and has resulted in informal task-sharing among the health workers. The knowledge and ability of CHEWs to perform basic clinical assessments, such as measure blood pressure is not in doubt. Nevertheless, there were divergent views by senior and junior cadres of health practitioners about CHEWs’ abilities in providing obstetric care. Similarly, there were concerns by various stakeholders, particularly the CHEWs themselves, on the regulatory restrictions placed on them by the Standing Order.

**Conclusion:**

Generally, the extent to which obstetric tasks could be shifted to community health workers will be determined by the training provided and the extent to which the observed barriers are addressed.

**Trial registration:**

NCT01911494

**Electronic supplementary material:**

The online version of this article (doi:10.1186/s12978-016-0216-y) contains supplementary material, which is available to authorized users.

## Plain English summary

This study shows how low-cadre health workers can be used to perform some of the duties of senior health care providers in order to reduce deaths of pregnant women as a result of preeclampsia. Information for this study was collected from different health care providers through group discussions with various categories of people across the community, health facilities and some policy makers. Result shows that one of the major challenges health personnel are facing is shortage of health personnel. Community health workers who were supposed to spend more time caring for women at the community level have remained at health facility. This enabled community health workers to learn and perform some of the tasks that were meant for senior cadre health personnel; for example, task like measuring of blood pressure and other clinical assessments. Though, this was the reality, health personnel did not have common agreement as to whether low cadre health personnel like CHEWs would be able to perform these and other basic tasks very well. One of the major obstacles which the CHEWs themselves pointed out was that they may not be able to accept some of the duties of senior cadre health personnel because of the Standing Order. The Standing Order is a document that guides the practice of health personnel in Nigeria. Therefore if the tasks of senior cadre health care provide are be shared by low-cadre health care provider, training must be considered and the challenge posed by the Standing Order should be addressed.

## Background

The dearth of health personnel in low income countries has attracted global attention. Among the top challenges being addressed are issues as to how health care services can be delivered in a more effective and efficient manner using available health personnel. Task-sharing is one innovative approach that makes this possible [1, 2]. It is the process which allows low-cadre and mid-level healthcare professionals to provide safely clinical tasks and procedures that would otherwise be restricted to higher level cadres [1, 2]. It has been used to improve access to contraceptives in Canada [2], HIV Care and ART services in Zambia [3], and in rural Malawi [4], and other health services in low and middle-income countries (LMIC) where there are widespread shortages of qualified health professionals [1–4]. This approach expands the responsibilities of low-cadre health personnel in an effort to best utilize available human resources [1, 2]. The shortage of health care providers is due to poor reform in the health system that could expand the number of trained health care workers, as well as address the “brain drain” phenomenon in Nigeria [5]. In 2006, the World Health Organization (WHO) estimated a global shortage of 4.3 million health workers, with poorer countries in the Global south particularly hard-hit [6].

Maternal mortality remains a grave concern globally with 210 maternal deaths per 100,000 live births in 2013 [7, 8]. Nigeria has one of the highest maternal mortality ratios at 560 per 100,000 live births [7]. The hypertensive disorders of pregnancy, including pre-eclampsia, are responsible for a large number of these preventable deaths. Although no antenatal remedy is available for pre-eclampsia, timely identification and management greatly decreases the likelihood of severe morbidity and death. Prompt identification of pre-eclampsia through reliable blood pressure and proteinuria measurement, emergency treatment with MgSO_4_ and antihypertensive agents, and timely referral enables many women and babies tof be saved.

Despite that Nigeria has one of the largest stocks of health workers in Africa, the density of nurses, midwives, doctors, and community health workers is too low to effectively deliver essential health services to the whole population. Inconsistent recruitment policies and the brain drain have led to a huge burden in health care delivery for the disproportionate number of employed health workers. Various cadres of health workers provide services in Nigeria. The Community Health Extension Worker (CHEW) provides primary health care services, it is expected that they spend 60 % of their time on community-based functions and 40 % on clinic-based functions. CHEW training is 2–3 years, and the curriculum is mainly focused on community diagnosis and treatment of minor ailment and diseases; assisting mid-level health workers in providing care at health facilities; and community outreach. There are no courses specifically dedicated to the hypertensive disorders of pregnancy in the CHEWs training curriculum; however, a course on reproductive health provides an overview of pregnancy and its complications. Objectives of the reproductive health course are to understand the concept of reproductive health and rights, including family planning, the process of pregnancy formation and development, the management of labour, and the care of the mother and child during puerperium. Other courses that could provide a foundation that might be relevant in the management of pregnancy hypertension include clinical skills and supervised clinical experience.

Within this context, it is important to investigate the potential for task-sharing to community health workers with specific reference to the provision of screening and detection of early signs of pre-eclampsia. The aim of this study is to identify the facilitators and barriers to the task-sharing of these services by the CHEWs in Nigeria.

### Description of study sites

This study was conducted in Ogun State, in southwest Nigeria; residents are predominantly Yoruba (See Table 1 for site description). The health indicators of Ogun State are reflective of the health indicators for Nigeria as a whole [9]. There are high levels of poverty, fertility and mortality.

**Table 1:** Study site characteristics

Nigeria characteristics	
Population	159,288,426
Size (Km^2^)	923, 768
Number of states	36
Number of geopolitical zones	6
Predominant language	Yoruba, Igbo, and Hausa
Predominant religions	Christianity and Islam
Ogun State characteristics	
Population	4,000,000
Size (Km^2^)	16,409
Number of local government areas	20
Predominant language	Yoruba
Predominant religion	Christianity
Local Government Area characteristics	
Cumulative population	469,271
Cumulative size (Km^2^)	1657
Number of clusters sampled	4
Number of wards	40

## Methods

This study combined qualitative and desk review methods to collect data from health personnel and health institutions. Through focus group discussions, in-depth interviews and document reviews, information was gathered regarding CHEW’s current level of competency in identifying and managing cases of pre-eclampsia. Competency assessments evaluated pre-service training and how that relates to the recognition of warning symptoms, screening, and treatment for pre-eclampsia. In addition, community members and various stakeholders were engaged to assess their support for additional task-sharing.

This study is part of a larger community-based trial evaluating the acceptability of community treatment for severe pre-eclampsia in Nigeria [10]. Seven focus groups discussion (FGD) were held with CHEWs (*n* = 71), three with male decision-makers (*n* = 35), six with community leaders (*n* = 68), and one with members of the Society of Obstetricians and Gynaecologists (*n* = 9). In addition, in-depth interviews (IDI) were conducted with the heads of the local government administration (*n* = 4), medical officers (*n* = 2), directors of planning (*n* = 4), and Chief Nursing Officers (*n* = 4). Interviewers were selected based on experience in the community, familiarity with the health care system, and qualitative research experience. Interviews were predominantly conducted in the local dialect (Yoruba), to allow deeper exploration of the participants’ experiences and feelings. Each FGD and IDI session was tape-recorded, translated, transcribed, and reviewed for consistency. Saturation was satisfactorily met from all stakeholder groups.

Qualitative data were translated into English by local researchers with fluency in Yoruba and English. All translations were reviewed by study investigators to ensure accuracy. Data were subsequently analysed using NVivo version 10.0 3 computer software. The analytical framework and coding structure were developed by the study principal investigators and data coder; however for consistency, all coding was performed by one individual. The majority of themes were identified a priori to represent community perceptions of pre-eclampsia; however a small number of additional themes emerged from the data through the coding process and were added to the structure at that time.

The Health Research and Ethics Committee of Olabisi Onabanjo University Teaching Hospital, Sagamu, Nigeria (OOUTH/DA/326/431), and the Clinical Research Ethics Board of the University of British Columbia, Vancouver, Canada (H12-00132), approved this study. Informed consent was obtained from all participants before the commencement of FGD or IDI session.

## Results

Demographic characteristics of discussants and respondents were collected during focus groups discussions on all participants; for details of participants see Tables 2 and 3. Christianity and Islam were the main religion, with a few followers of traditional faiths. All participants were married; with most having at least one child and some up to 7 children.

**Table 2:** Focus group discussion participants

#	Groups	Local Government Area	Number of participants
1	Community Health Extension Workers	Sagamu (Ogijo Axis)	12
2	Community Health Extension Workers	Sagamu (Ogijo Axis)	12
3	Community Health Extension Workers	Yewa South	12
4	Community Health Extension Workers	Yewa South	12
5	Community Health Extension Workers	Imeko-Afon	12
6	Community Health Extension Workers	Imeko-Afon	12
7	Community Health Extension Workers	Remo North	11
8	Community and Religious Leaders	Sagamu (Ogijo Axis)	10
9	Community and Religious Leaders	Yewa South	12
10	Community and Religious Leaders	Imeko-Afon	12
11	Community and Religious Leaders	Remo North	12
12	Community and Religious Leaders	Remo North	10
13	Community and Religious Leaders	Sagamu (Ogijo Axis)	12
14	Male Decision-Makers	Yewa South	12
15	Male Decision-Makers	Imeko-Afon	12
16	Male Decision-Makers	Remo North	11
17	Members of the Society of Obstetricians and Gynaecologists	Nigeria	9

**Table 3:** In-depth interview participants

#	Participants	Local Government Area
1	Head of Local Government Administration	Sagamu (Ogijo Axis)
2	Head of Local Government Administration	Yewa South
3	Head of Local Government Administration	Imeko-Afon
4	Head of Local Government Administration	Remo North
5	Chief Nursing Officer	Remo North
6	Chief Nursing Officer	Sagamu (Ogijo Axis)
7	Chief Nursing Officer	Yewa South
8	Chief Nursing Officer	Imeko-Afon
9	Director of Planning	Sagamu (Ogijo Axis)
10	Director of Planning	Yewa South
11	Director of Planning	Imeko-Afon
12	Director of Planning	Remo North
13	Medical Officer of Health	Yewa South
14	Medical Officer of Health	Remo North

### Human resource constraints

Discussions with community health workers demonstrated that availability of personnel was a major challenge. In most regions, few health workers are available in health centres, particularly in rural communities. Community health workers expressed their worries about the challenges they faced due to human resource constraints. One of them declared that, if they *“have more workers, it would make the work easier than how it is now*”. A director of planning described the inadequate number of health personnel in primary health centres throughout the state:

*We don’t have enough health care workers, for example, maybe they are supposed to have three shifts in a health care facility and for each shift, maybe they are supposed to have a nurse, a CHEW and a health care attendant on duty so there are supposed to be nine of them available for the three shifts and some others so let us say twelve there is no health care facility that one would visit and find twelve health care workers there at any point in time, because even the local government doesn’t have the money to pay such salaries they wouldn’t have the money to pay their salaries and that is the major problem.*

*In-depth interview with the Director of Planning, Yewa South*

An in-depth interview with one Chief Nursing Officer revealed that only 33 CHEWS and 29 nurses/midwives were available for deployment in the entire local government area, a number that is far below the requirement for standard practice.

The lack of health personnel is not only due to a scarcity of trained health workers, but also the inability of local government agencies to effectively recruit and retain these workers. One local government officer expressed that the government “*can co-opt some of them”;* even if it is based on contract appointment. Institutions often lack the financial resources to employ the required community-based health care workers. This invariably has created a set of health workers who serve as private consultants at the community level. These private consultants frequently provide some basic obstetric services beyond their scope as outlined in the Standing Orders. The Standing Order is a documented instruction that guides community health workers how to administer specified medicines or controlled drugs. Similarly, community health workers employed at primary health care centres often perform tasks beyond their Standing Order.

### Informal task-sharing

Currently, CHEWs’ work is based predominantly at the health centre. Therefore, they often care for cases beyond their designated scope. These tasks include attending deliveries and basic obstetric care. The narrative below provides insight into the practice of CHEWs:

*Sometimes a person can be on duty, the same person would be the one to help the pregnant woman to push the baby out… she would quickly rush down to receive the baby herself*

*Focus group discussion with CHEWs, Sagamu (Ogijo Axis)*

There is an informal task-sharing arrangement between nurses, midwives and community health workers. In most instances, nurses and midwives were not willing to stay in rural communities when posted. Invariably therefore, some of the tasks assigned to nurses and midwives were carried out by CHEWs. One Chief Nursing Officer alluded to the concerns of the current situation where community health workers have focused on facility-based care at the detriment of home-based care.

*We had a […] time when nurses were being grossly inefficient, so our CHEWs have drawn back to health facilities and so most of the home-based cares has been really low […] A situation in which I don’t have anybody to attend to my clients in the health facility…and you want me to release the few there to go and be engaged in home care it’s not that feasible but then if you are able to do it, it would reduce the load on us seriously and of course, we would be able of catch most of this ailments as early as possible.*

*In-depth interview with medical officer, Yewa South*

The sentence above demonstrates the support for health workers posted in the community; however, such a shift is not currently felt to be feasible due to the shortage of workers. The involvement of CHEWs in obstetric care has provided them essential hands-on experience.

### Task-sharing for obstetric care

The existing informal task-sharing arrangements have integrated CHEWs in basic obstetric care. Despite informal task-sharing among health workers, there were reservations among senior health personnel as to whether additional redistribution of responsibilities would be appropriate. A senior medical doctor viewed that scaling up the skills of CHEWs may lead to a greater demand for their services, especially in private facilities and urban centres, leading to service drift and additional shortage of CHEWs.

*If you train [Community Health] Extension Workers to a higher level whereby they can now comfortably handle preventing pre-eclampsia and then handling deliveries very well, how are we sure that what happened to the midwives won’t happen to them? Their value can rise and they may no longer be available.*

*Focus group discussion with members of the Society of Obstetricians and Gynaecologists of Nigeria*

Nevertheless, many expressed support for task-sharing under the condition that CHEWs receive relevant training in obstetric care.

*I think these Extension Workers could be trained, because you see we don’t have enough manpower to put doctors in all the health centres […]. So even if they are trained to be able to check the blood pressure properly, given certain value at which it is regarded as not normal and refer early, that will go a long way in this community prevention of the pre-eclampsia.*

*Focus group discussion with members of the Society of Obstetricians and Gynaecologists of Nigeria*

### Blood pressure and proteinuria measurement by CHEWs

Measurement of blood pressure is central to the diagnosis of pre-eclampsia, it was clear from all participants in focus groups and interviews, that CHEWs can reliably measure blood pressure. It was mentioned in all eight focus groups with CHEWs and all four interviews with Head CHEWs that they are currently measuring blood pressure and assessing basic vital signs. CHEWs as well as their supervisors were confident in their ability to take blood pressure: “*even the junior CHEW…the least among the rank of CHEWs […] knows how to measure a patient’s blood pressure accurately”.* Unlike blood pressure, proteinuria assessment was rarely performed during antenatal screening by CHEWs. Faced with the challenge of lack of equipment, CHEWs relied heavily on observed symptoms associated with pre-eclampsia and eclampsia to assess risk.

### Pre-eclampsia management by CHEWs

CHEWs were thought to have the ability to manage uncomplicated obstetric cases at the community level; however, there were divergent views regarding their ability to provide services for complex cases such as pre-eclampsia. Some of the senior health personnel interviewed worried about the risks involved in committing such care to CHEWs. One administrative head shared the view that some obstetric tasks could be shifted to CHEWs if they are well trained:

*In the first instance we are not having enough of doctors, we are not having enough nurses and definitely it is the CHEWs that we have in relative large number when compared to these other officers […]. They will need more training on the job in order to assist them […] in the care of the pregnant women with elevated blood pressure. They will need additional training especially in the form of workshops and seminars, I’m talking of very serious clinically based training.*

*Interview with the head of Imeko-Afon Local Government Area*

Focus groups and interviews identified barriers to task-sharing for community health workers. Many of these barriers relate to legal restrictions outlined in the Standing Order, and worries related to insufficient training. There were concerns by various stakeholders, particularly the CHEWs themselves, regarding regulatory restrictions. References were frequently made to the fact that they are legally protected only if they adhere to the instructions in the Standing Order. Although CHEWs were permitted to provide immunizations to children in their homes, they were not allowed to give other types of injections outside the clinic without supervision.

*If we see that a pregnant woman has high blood pressure we would call on our doctor and he/she would go and attend to the pregnant woman, and sometimes if the doctor is not around we would take our Standing Order we would check for how to manage the pregnant woman’s condition and that is what we would do, if the Standing Order says we should prescribe drugs for the pregnant woman we would prescribe drugs for the pregnant woman*

*Interview with the Head CHEW, Remo North*

For cases of pre-eclampsia and eclampsia, the CHEW Standing Order indicates that intramuscular diazepam should be given prior to referral.

There were conflicts amongst health workers regarding turf protection. Some nurses and midwives felt that CHEWs should not be given the authority to “treat” obstetric cases, such as pre-eclampsia. CHEWs require the administrative support of nurses and midwives for task-sharing to be successful.

A large number of respondents were of the view that sophisticated facilities and equipment were required to monitor patients receiving MgSO_4_, which is unavailable in most health centres. This underscores why some health personnel believed it is not feasible.

*We know that the gold standard is magnesium sulphate, but you know the problem associated with that, monitoring level and so on and so forth. But then the diazepam that can be used without much monitoring… but they are not available, so what I am saying is that we lack infrastructure, there are no staff, common drugs; sedatives like diazepam is lacking.*

*Focus group discussion with members of the Society of Obstetricians and Gynaecologists of Nigeria*

In addition, there was a great deal of consensus as to the scarcity of commonly used medications in obstetric practice. These stock outs are prohibitive to any task-sharing of obstetric care.

Most opinion leaders expressed reservations about male CHEWs involvement in obstetric care in the home. One of the male decision-makers shared his view on this subject:

*Her husband might be thinking that the health care worker is trying to play tricks to marry his wife. The health care worker wants to use that avenue to woo his wife, we have seen cases whereby a man would disguise as a woman and would to visit a family […] not knowing that it was a man disguised as a woman, the man would go into the room with [another man’s] wife and sleep with her […]. This is one of the reasons why a man wouldn’t want health care workers to come to his house. The man could be thinking that the health care work has interest in his wife.*

*Focus group discussion with male decision-makers in Yewa South*

There is distrust for male CHEWs handling obstetric issues, these feelings tend to be both held and expressed by men. While this was the majority opinion, a few expressed confidence in male CHEWs’ ability to provide obstetric care.

## Discussion

This qualitative study demonstrates the huge prospect of task-sharing among nurses/midwives and community health workers in the detection of early signs of preeclampsia. Literature from Africa and Canada, demonstrate how task-sharing can be successfully utilized to address complex problems of health care delivery. Currently, a randomized, controlled, non-inferiority trial for task-sharing for the care of severe mental disorders in low income countries is is ongoing [11]. An argument for task-sharing of some obstetric care to CHEWs is due to health personnel inadequacies [2] and as an attempt to drive the efforts in the reduction of maternal mortality in Nigeria [7, 12]. Task-sharing among community health workers could improve health indicators. There were some genuine concerns regarding service and the drift of personnel among CHEWs once they have acquired obstetric skills. This could be countered by improving the welfare package of health workers and ensuring that CHEWs are being consistently trained to fill existing vacancies.

Human resource constraints inhibit community health workers from performing their community-based functions [13]. This may have created a gap between CHEWs and the community. With a higher CHEW concentration in facilities, they have continuous practice of health care delivery under the supervision of nurses and midwives which has led to some degree of skill transfer. This transfer of skills is evident in the type of cases they successfully attend to. It is imperative to formalize the acquired skills, to enhance their abilities and provide the leverage required for task-sharing. Of course, this will also require a re-visit of their curriculum ultimately and an amendment of the Standing Order to address the legal implications. Turf protection can be a concern; the potential for turf protection between nurses, midwives and CHEWs is very high. Since CHEWs are expected to be sharing some of the tasks currently being handled by nurses and midwives, the prospect for a smooth transition is low [14] without formalised approach.

The current training, recruitment and deployment strategy has created a contradiction in the roles of community health workers because it systematically detaches them from the community. Although, CHEWs are expected to deploy 60 % of their time in the community and 40 % in the facility, the current arrangement inhibits them from direct engagement with the community. This, therefore, calls for a re-examination of the recruitment and deployment policy of CHEWs. Annually, scores of CHEWs are being trained but only few are being employed. The non-recruitment which has led to human resource constraint among community health workers may have resulted in a shift to private practice by some qualified CHEWs. Invariably, the policy to restrict employment of community health workers may have adverse effects on the health of women and the country at large. In other words, when the huge numbers of unemployed, but qualified community health workers are employed, the resulting gains will far outweigh its loss.

## Conclusion

Supplementary training, in addition to the facility-based experience CHEWs have had, could help them to adopt new responsibilities and help to ensure efficient delivery of obstetric care in Nigeria. Of importance is the re-training of community health workers to enhance content regarding obstetric care which has not been included in the Standing Order.

### Limitations of this study

This study is essentially qualitative and as such findings may not apply to other ethnic groups outside the Southwest geo-political zones where the Yoruba are the predominant ethnic group.

## Additional file

Additional file 1: Reviewer reports. (PDF 161 kb)

## Abbreviations

CHEW: Community health extension workers
FGD: Focus groups discussion
IDI: In-depth interviews
LMIC: Low and middle-income countries
WHO: World Health Organization.
