# Supplementary material for: Human resource constraints and the prospect of task-sharing among community health workers for the detection of early signs of pre-eclampsia in Ogun State, Nigeria
Source: Reprod Health. 2016 Sep 30;13(Suppl 2):111. doi: 10.1186/s12978-016-0216-y (PMC5056470; doi:10.1186/s12978-016-0216-y)
Supplement: Additional file 1: — Reviewer reports. (PDF 161 kb) [file 12978_2016_216_MOESM1_ESM.pdf]

Referee's comments to the authors– this sheet WILL be seen by the author(s) and published with the article

Title	Human resource constraints and the prospect of task-shifting among community health workers for emergency management of pre-eclampsia in Nigeria
Author(s)	David O. Akeju, Marianne Vidler, Sotunsa, JO, Osiberu, MO, Orenuga, EO, Olufemi T. Oladapo, Adepoju, AA, Rahat Qureshi, Diane Sawchuck, Olalekan O. Adetoro, Peter von Dadelszen, Olukayode A. Dada and the CLIP Nigeria Feasibility Working Group
Referee's name	Bukola Fawole

When assessing the work, please consider the following points, where applicable:

1. Is the question posed by the authors new and well defined?
2. Are the methods appropriate and well described, and are sufficient details provided to replicate the work?
3. Are the data sound and well controlled?
4. Does the manuscript adhere to the relevant standards for reporting and data deposition?
5. Are the discussion and conclusions well balanced and adequately supported by the data?
6. Do the title and abstract accurately convey what has been found?
7. Is the writing acceptable?

Please make your report as constructive and detailed as possible in your comments so that authors have the opportunity to overcome any serious deficiencies that you find and please also divide your comments into the following categories:

- Major Compulsory Revisions (which the author must respond to before a decision on publication can be reached)
- Minor Essential Revisions (such as missing labels on figures, or the wrong use of a term, which the author can be trusted to correct)
- Discretionary Revisions (which are recommendations for improvement but which the author can choose to ignore)

Where possible please supply references to substantiate your comments.

When referring to the manuscript please provide specific page and paragraph citations where appropriate.

General comments:
The manuscript addresses task-shifting as an approach to increasing access to health care services. Is it possible that opposition to this concept described as turf protection can be mitigated by adopting the terminology task-sharing?

Major compulsory revisions:

None

Minor essential revisions:

In the Abstract**,** do the Authors mean Conclusion rather than Discussion?
In the Background section, the first reference should be cited as 1, not 3.
Under Methods, the first sentence is rather ambiguous.
It is essential to describe and confirm the consent procedure for the FDGs and IDIs.
Results: Ín almost all the primary health centres, the minimum number of health care providers was not met'. The Methodology as presented does not provide any basis for this statement.

Continued:References
Authors need to review the format for the references. Surname usually precedes the initials.

Discretionary Revisions
A brief explanation of Standing Orders is warranted. Is it the same as job description?

Table 1: One expects that the predpminant language in Ogun State in Nigeria should be Yoruba.

Table 3 should be reviewed.

Referee's comments to the authors– this sheet WILL be seen by the author(s) and published with the article

Title	Human resource constraints and the prospect of task-shifting among community health workers for emergency management of pre-eclampsia in Nigeria
Author(s)	David O. Akeju, Marianne Vidler, Sotunsa, JO, Osiberu, MO, Orenuga, EO, Olufemi T. Oladapo, Adepoju, AA, Rahat Qureshi, Diane Sawchuck, Olalekan O. Adetoro, Peter von Dadelszen, Olukayode A. Dada and the CLIP Nigeria Feasibility Working Group
Referee's name	Jack Moodley

When assessing the work, please consider the following points, where applicable:

1. Is the question posed by the authors new and well defined?
2. Are the methods appropriate and well described, and are sufficient details provided to replicate the work?
3. Are the data sound and well controlled?
4. Does the manuscript adhere to the relevant standards for reporting and data deposition?
5. Are the discussion and conclusions well balanced and adequately supported by the data?
6. Do the title and abstract accurately convey what has been found?
7. Is the writing acceptable?

Please make your report as constructive and detailed as possible in your comments so that authors have the opportunity to overcome any serious deficiencies that you find and please also divide your comments into the following categories:

- Major Compulsory Revisions (which the author must respond to before a decision on publication can be reached)
- Minor Essential Revisions (such as missing labels on figures, or the wrong use of a term, which the author can be trusted to correct)
- Discretionary Revisions (which are recommendations for improvement but which the author can choose to ignore)

Where possible please supply references to substantiate your comments.

When referring to the manuscript please provide specific page and paragraph citations where appropriate.

General comments:
A relevant paper

Major compulsory revisions:

Reconsider the title. Suggestion: Human resource contrains and the prospect of task-sharing amoung community health workers for the detection of early signs of pre-eclampsia

Minor essential revisions:

State limitations of this approach

Discretionary revisions:
